# Users’ Experience of Public Cancer Screening Services: Qualitative Research Findings and Implications for Public Health System

**DOI:** 10.3390/bs14020139

**Published:** 2024-02-16

**Authors:** Maria Florencia González Leone, Anna Rosa Donizzetti, Marcella Bianchi, Daniela Lemmo, Maria Luisa Martino, Maria Francesca Freda, Daniela Caso

**Affiliations:** Department of Humanities, University of Naples Federico II, 80133 Naples, Italy; mariaflorencia.gonzalezleone@unina.it (M.F.G.L.); marcella.bianchi@unina.it (M.B.); daniela.lemmo@unina.it (D.L.); marialuisa.martino@unina.it (M.L.M.); fmfreda@unina.it (M.F.F.); caso@unina.it (D.C.)

**Keywords:** cancer screening, cervical screening, breast screening, colorectal screening, prevention, public health

## Abstract

Following the One Health approach, designing multidimensional strategies to orient healthcare in promoting health and preventive processes has become paramount. In particular, in the prevention domain, cancer screening attendance is still unsatisfactory in many populations and requires specific consideration. To this end, following a research-intervention logic, this study aims to investigate the experiences and meanings that users of public cancer screening services associate with prevention, particularly participation in the screenings. The experiences of 103 users (96 females; M_age_ = 54.0; SD = 1.24) of public cancer screening programs in the Campania region (Italy) were collected through interviews. The data collected were analysed following the Grounded Theory Methodology, supported by the software Atlas.ti 8.0. The text material was organised into eight macro-categories: Health and Body; Relationship with Cancer and Diseases; Health Facilities and Health Providers; The Affective Determinants of Cancer Screening Participation; Partners and Children; Physical Sensations and Emotions in the Course of Action; Protective Actions; Promotion and Dissemination. The core category was named Family and Familiarity. Respondents perceived prevention as an act of care for the family and themselves. Our findings support a shift from the idea of taking care of personal health as an individual matter toward considering it as a community issue, according to which resistance to act is overcome for and through the presence of loved ones. The results of this study contribute to a deeper understanding of the perspectives of southern Italian users on participation in cancer screening, and provide important insights to guide future actions to promote these public programmes based primarily on the emerging theme of family and familiarity related to screening programs.

## 1. Introduction

Cancer is a steadily growing health issue worldwide, and was the cause of almost 10 million deaths in 2020 [1]. However, more than 40% of cancer deaths could be avoided annually by eliminating environmental, metabolic, or behavioural risk factors that increase the likelihood of contracting the disease. That would result in 4.45 million avoidable deaths, almost 2.9 million of which would affect men with risk behaviours such as smoking cigarettes, alcohol consumption, and a poorly varied diet [2]. Although the adoption of healthy behaviours and habits is an important primary prevention approach to reduce the risk of cancer, screening programs are currently the main tool for obtaining an early diagnosis, which provides the opportunity to intervene in the early stages of the disease, resulting in a drastic reduction in mortality and severity of the illness [2]. Coherently, the European Code Against Cancer [3] identifies twelve rules for effectively combating this disease at the individual level. These rules include both primary prevention actions for adopting a healthier lifestyle and removing everyday bad practices (such as healthy eating, physical activity, not smoking, etc.), as well as secondary prevention actions such as regularly participating in cancer screenings for early detection.

The Italian government invests plenty of economic resources into cancer prevention. Indeed, the survival rate in this country is higher than the European average, with equal average incidence [2]: for example, Italians aged between 50 and 74 years who in 2019 declared to have had a colorectal cancer screening in the previous two years were 36% compared to 33% in the EU. In particular, the efficacy of three screening programs—for colorectal, breast, and cervical cancer—has been validated, and these programs are aimed at specific population groups identified based on gender and age, to whom periodic examinations are offered free of charge. Specifically, participation in colorectal cancer screening is recommended for men and women aged 50–74 every 2 years; breast screenings are recommended for women aged 45 or 50 to 69 years at 2-yearly intervals; and cervical screenings are recommended for women aged 25–64 every 3 years.

Although the overall Italian situation may suggest a reassuring picture while compared to the European rates, there are significant internal territorial differences: in 2019, the percentage of people who reported having been screened for colorectal cancer in the previous two years in the centre-northern Italian regions was six times higher than in the southern regions, with Campania ranking third to last [4]. Similarly, such disparity is also observed for both cervical (70.2% vs. 16.1%) and breast (75.8% vs. 11.8%) screenings [5]. Moreover, for all considered cancer screenings (breast, colorectal, and cervical), the attendance rate is higher for people with a higher education level and for those who did not report economic difficulties [4].

To tackle this issue, the Campania Region has launched the ‘Mi Voglio Bene’ program, which supports citizens’ participation in public prevention services by offering women and men resident and domiciled in the region specialised and free diagnostic assistance for preventing breast, colorectal, and cervical cancer. The values that inspire the regional strategies in health and prevention programming are equity, integration, and citizens’ participation, based on the awareness that different health states are closely correlated to different living conditions and, ultimately, to well-being. Thus, free screening programs are organised to reduce socio-economically based inequalities, per the idea that health is a basic human right for all. Health, considered a social and not a private matter, must be protected with integrated efforts: this principle, affirmed by the World Conference on Women [6] in the fourth action program, emphasises the need to look at women’s and men’s health as an overall state of psycho-physical well-being, which should be protected through targeted and differentiated initiatives based on the specificity of the audience. Despite the existence of this program, statistics show that adherence to cancer screenings in Campania is still rather scarce [7], and significantly lower than in other Italian regions, particularly the northern ones.

### Objectives

This study aims to investigate the meanings that users of public screening services attribute to the general topic of prevention, and specifically to their participation in free cancer screening programs in a territorial context where the participation rates are particularly low, namely the Italian Campania region.

By adopting a bio-psycho-social perspective on health [7]—which emphasises the importance of simultaneously considering the biological, psychological and social levels—the topic of cancer prevention needs to be analysed in a broader context in which lifestyle behaviours, quality of life, health policies, health care, and early detection are some of the many factors that intervene to create a single outcome. Therefore, in order to design a multidimensional strategy to drive increased participation rates in cancer screenings, exploring the perspective and experiences of users of these services acquires crucial importance, with a focus on the interactions between health professionals and patients in various care settings [8,9]. In this regard, qualitative research methodologies offer an important tool for developing this type of knowledge, allowing the exploration of the experiences and networks of meanings that people generate around a particular theme [10,11,12]. Moreover, among the various qualitative approaches, the Grounded Theory Methodology (GTM) [13,14,15] sheds light on social phenomena not pre-ordained by pre-existing theoretical paradigms [16].

## 2. Materials and Methods

### 2.1. Recruiting of the Participants and Tools

From May to July 2022, in-person interviews were conducted by a Ph.D. in Psychology at the healthcare facilities where the screening exam was attended. More specifically, after the screening, the user was asked to meet the interviewer, who explained the purpose of the research and invited them to participate in the study. After signing the informed consent, the participants also authorised the audio recording of the interview, whose average duration was 15 min (minimum: 07 min; maximum: 25 min). The interview grid and the entire research layout were approved by the Ethics Committee of the University of Naples Federico II (prot. n° 16/2022). The interviews followed a dialogical approach, where (i) prescribes the interviewers to not be directive, as they should not interrupt the stream of the interviewee’s thoughts, but let it flow as freely as possible; (ii) guarantees specificity; (iii) is inclusive, conferring the same importance to all the data, even if contraposed to what other interviewees reported; and (iv) explores all facets of the interviewee’s experience. This approach does not include predetermined questions. Rather, employing a focused narrative interview [17], the interviewer followed a guide outlining the main issues to be addressed. The grid encompassed the following areas to be explored: (1) aspects of the self involved in the representation of prevention; (2) body, risk perception, and health; (3) the role of health facilities and personal resources in managing one’s health; (4) specificity of participation in cancer screenings; and (5) future perspectives.

### 2.2. Data Analysis

Data analysis was carried out following the Grounded Theory Methodology (GTM) [13,14,15], supported by Atlas.ti 8.0 software [18]. Preliminary, all recorded interviews were transcribed verbatim. Then, the research team coded the textual material using a bottom-up approach in a three-step process, according to the level of meaning abstraction. All interviews were double coded. First, the text was open-coded independently by each researcher. All codes were then organised into code groups (categories). Then, the axial coding was performed to understand the proposed meanings of the texts better, and the categories were arranged into more abstract macro-categories. The last stage (selective coding) involved the final data conceptualisation by identifying a core category. At all stages of the analysis process, the research team discussed the meanings capitalising on the reflexive skills of each member involved.

## 3. Results

### 3.1. Participants’ Characteristics

A total number of 103 cancer screening service users (7 males and 96 females) aged 25–70 years (M = 54.0; SD = 1.24) participated in the present study. Participants were recruited and interviewed after having attended one of the three cancer screenings offered free of charge in the Campania region (colorectal, breast, and cervical). By employing a purposive sampling approach, the inclusion criteria for participation in the study were (i) being a user of one of the seven public Local Health Departments in Campania (three for the province of Naples, one each for Benevento, Avellino, Caserta, and Salerno) and (ii) having recently performed at least one of the three screenings at a public facility. The participants had various work statuses (Table 1). About half of them had a family history of cancer (53.4%), and 55.3% relied only on public facilities for cancer screening exams.

The size of the sample was determined following the principle of data saturation, i.e., the authors decided to stop the collection of new interviews when the new data ceased to generate new insights or themes. Following 103 interviews, the research team established that sufficient depth and breadth of information had been reached, with little to no new information emerging.

### 3.2. Categories and Macro-Categories

The research participants described their experience, enabling researchers to reach 343 codes, 21 categories, 8 macro-categories, and a core category.

The core category identified was named Family and Familiarity, reflecting the idea of prevention considered as an act of caring for others. Cancer, disease, and risk appear to be strictly associated with two words: family history. The fears, representations, anxieties, and behaviours enacted are linked to participants’ own accounts of events, losses, and illnesses in the family. These experiences have the power to activate a subjective and individual way of experiencing prevention in general and cancer screenings in particular.

This can be deduced from the categories and macro-categories that emerged, which we will describe below (Table 2).

#### 3.2.1. Health and Body

When confronted with grid questions, the participants associated health with elements more related to the body rather than the mind. The body is referred to as a collector of experiences, as an enemy when it comes to body weight and nutrition, as a target, and as the main site of shame and embarrassment when facing the screening exam. The disease for the women interviewed is not only linked to a diagnosis, but also to a body that does not fit the proposed models, with excess weight and signs of ageing, unhealthy food, and lack of physical activity.

This macro-category comprises 2 categories: Representation of health and Body representation.

The research participants describe their health as the most important element of life, as being happy would be impossible without it:

*“Let’s say it’s the first thing, that’s it. In my opinion, it’s more important than everything, and people should really prioritise it, that’s it”*

(woman, 50 years old, employed, breast screening).

*“For me, health is the most important thing and should always be our first thought. Check-ups are important, and I have my medical exams as far as I can. For instance, every year, I have my tests, you know, starting with the smallest things. These are the basics!”*

(woman, 51 years old, housewife, breast screening).

*“That’s just what I was telling the doctor before: as I watch “Buongiorno Benessere” on TV every day, for me, health equals well-being! I mean it is important to be healthy, so you are also happier and can give more to others”*

(woman, 65 years old, retired, colorectal screening).

Health for many respondents is either a desire to be achieved because they do not have it, or something to be offered because it is considered an opportunity for taking care of others:

*“For me, it’s a wish... In 2014 I had surgery for melanoma, then in 2015 another one for colorectal cancer and in 2017 for liver metastasis. So, for me, the word health is a nice wish…”*

(woman, 57 years old, employee, breast screening).

*“Because if you are sick, the children, the grandchildren, the husband... let’s say that the woman is the person who manages a little bit of everything in the family (...). So, my children say: ‘Mom, you have to take care of yourself for us… We need you!’. So, sometimes you take care of yourself, just for them”*

(woman, 69 years old, unemployed, breast screening).

The female body emerges in our research as controversial and stigmatised. Not only are the women we met concerned for their physical health, but they also express the discomfort experienced with their bodies:

*“With my body, I have quite a conflicting relationship… I mean, I was happy to come here today for the screening, but if I had to take the initiative myself, perhaps I would not have done it. But I am still happy when someone cares about me regularly—I have to admit it—I am glad to be invited to come and have the exam”*

(woman, 58 years old, lawyer, cervical screening).

*“Eh…with my body… let’s say it’s controversial. About the aesthetic, well, I think that maybe everyone has something they don’t like about their body. So, with the prevention, it could be similar, maybe one should do some prevention, do some treatment… just do something, in short”*

(woman, 45 years old, unemployed, cervical screening).

While voicing their feelings about their bodies during screening, some women interviewed expressed embarrassment. They reflected on the fact that this may be a consequence of cultural taboos that are still evident in the medical field:

*“We are subjected to various taboos. For instance, let’s talk about vaginal health… Oh my, what did you say! Today, I still see strong fatigue in people—maybe also due to my job—when they should use terms like menstruation... It’s somewhat a taboo. It seems like saying something unbelievable, like the tampon… what is it? […] That’s something that upsets me. In fact, for me, that’s the problem, there are so many indirect ways of naming menstruation....”*

(woman, 33 years old, cultural mediator, cervical screening).

The body in women’s minds is strictly related to hormonal changes, which in turn generate changes in the way they experience their bodies and accept themselves:

*“I’m in menopause. I have headache problems, problems with my period. I haven’t had my period for a year now, but all my bones ache, my arms… I feel bad about myself”*

(woman, 55 years old, unemployed, breast screening).

*“Hopefully, this situation will end as soon as possible. I’m also trying to take some supplements that can help me with both the bloating and the mood because menopause consists of that awful thing: you go from a state where you are happy and content to a state of prostration, and you want to cry for no reason. The worst thing is the need to throw away all the clothes you’ve worn all your life, and that maybe you were delighted to buy, and for buying them you’ve even made sacrifices… Perhaps you’ve got a nice pair of trousers, then you try them on and they don’t go up… In short, it was a hideous experience, and it’s still like that… It’s awful”*

(woman, 54 years old, office worker, breast screening).

While the body does not appear at all in the narratives of the men we interviewed, it emerges disruptively in the women’s discourses as a form of complaint of a major affliction.

*“I don’t like looking at myself in the mirror. I don’t feel myself anymore. I’ve put on weight, swollen, and taking cortisone for six months, It’s been 15 days since I finished, but I’ve never felt so swollen”*

(woman, 46 years old, housewife, cervical screening).

*“I don’t have a good relationship with my body because I basically don’t like myself, and it’s because I’m clearly overweight; that’s something people always tell me”*

(woman, 61 years old, employed, cervical screening).

#### 3.2.2. Relationship with Cancer and Other Diseases

This macro-category was created to catch the narrative style of the interviewees. Facing questions about whether or not to adhere to screening, participants’ answers did not regard the type of medical intervention, the bureaucratic aspects of the system, or the objective issues of screening. Instead, their answers corresponded to narratives of events from family friends or close people about cancer. This suggests that it is the events experienced in the past that give meaning and significance to the screening exam, making the individual ways of accessing even more subjective. People do not evaluate the service for the service itself, but for its relation to previous experiences.

This macro-category comprises three categories: Family stories, Individual stories, and Stories of loved ones.

Our data show how much interviewees’ representations of and attitudes toward health prevention and cancer screening programs in Campania are directly influenced by the previous experiences that themselves, their family members and—generally—people they know and love had with cancer and other diseases. Thus, the stories of other people that come out through our participants’ words seem quite determinant in their actual preventive actions or in their tendency to avoid it:

*“As I said before, I lost my brother after my mum, and then immediately also my father, not even a year after my mum. My brother died of gastric cancer. It was really invasive. My brother suffered so much, even more than my mum and dad. First, it came out on his leg, then on his face… It was growing everywhere. It’s been six months since... Let’s say since December. We really didn’t understand anything. That’s why I’m here...”*

(woman, 50 years old, office worker, breast screening).

*“I think, here’s an example of my sister, who had never had a mammogram. At one point, she looked at her breast, saw this withdrawn nipple, immediately had an ultrasound, I mean, a mammogram, and suddenly had to have the breast removed”*

(woman, 65 years old, retired, colorectal screening).

Those who were invited to have the screening and subsequently attended it stated they were prompted by a recent loss in the family or the discovery of a loved one’s cancer:

*“I am here because of familiarity. My father died of colon cancer, and my brother of leukemia, the blood cancer”*

(woman, 69 years old, teacher, colorectal screening).

*“In my family, it happened that in January an uncle of mine died with lung cancer, and he had never had anything before, so this made me think about it...”*

(woman, 55 years old, unemployed, cervical screening).

The fears, the risk perception, and the idea of health and illness are richly saturated with experiences, memories, and pains for our interviewees. In this sense, the objective components of these representations tend to disappear in favour of a complex system of subjective associations that deeply interfere with the way of experiencing prevention in general and cancer screenings in particular:

*“It is preferable to do the screening immediately to avoid later problems. It’s also because I have seen so many cases of friends who practically had big problems… A short time ago, a 51-year-old friend died of breast cancer, she had had surgery two years before, and maybe the surgery wasn’t well-done because first she had chemo and everything was fine, she also had her check-ups, and everything was fine, but then, within two or three months... So, yes, prevention is a very good thing, but there are cases in which these diseases are so insidious that...”*

(woman, 66 years old, pensioner, breast screening).

*“A 45-year-old friend of mine realised she had cancer precisely because she attended a prevention exam, and today she is fine. For me, not doing prevention is risky, and it is a form of cowardice because often people do not want to know, but we should face it”*

(woman, 63 years old, trader, breast screening).

#### 3.2.3. Health Facilities and Health Providers

While the previous macro-category encompassed respondents’ responses regarding cancer screenings that mainly focused on individual experiences, this macro-category included the practical considerations that lead participants to chose a certain facility for their screening exams: waiting for the reservation, reception of operators, the quality of technical machines, the hygiene of the facility, the staff, the preference for public or private healthcare. The collected data suggest that the choice of a certain facility does not only rely on its geographic location, which does not directly determine adherence or nonadherence to the screenings.

This macro-category includes three categories: Positive aspects, Negative aspects, and Public vs. private healthcare.

While describing their experience with cancer screenings, participants identify the role of the health facilities they referred to, which decisively affected the choice of doing it or not, as well as the intention of coming back in the future. In this regard, their narratives highlight both positive and negative aspects. As for positive elements, participants reported various aspects of the experience with the facility: the ease of booking, the presence of welcoming medical staff, and their capabilities to provide correct information about the screening procedure. For example, in the case of colorectal screening, many pinpointed receiving clear information on how to keep the kit until the delivery as particularly important. In addition, other determining factors were the opportunity to quickly reach the location (e.g., living close by) or even to still access screening services in poorly connected areas through the organisation of ad hoc solutions, such as travelling campers:

*“It was advantageous. These initiatives they organise for women’s health… like these trucks on the street where you can access screening for free. Maybe they should do it even more often so that when a person finds them on the street, they do not need to queue, and maybe, in this way, they don’t get discouraged and just do it”*

(woman, 58 years old, employed, breast screening).

*“Because if I had wanted to do it on my own, I would have had to go to the doctor to get the prescription, then collect the sample...Instead, they did everything themselves, booking it was effortless. I just had to do the test, so I brought it here, and it was straightforward”*

(woman, 54 years old, lecturer, breast screening).

On the other hand, the negative aspects described by our interviewees mainly consist of the waiting time for the results, which is perceived as excessively long, and increased the levels of anxiety and concern. Other cited elements were the perception of a lack of empathy among the medical staff, the inadequacy of mammography equipment, the lack of flexibility in the opening hours of the structures, the lack of car parking near the facility, and the age restrictions for free access to the cancer screenings:

*“The only problem [of attending screenings in public facilities] is the opening hours of the facilities, so you have to use these time slots where you maybe have to come there from work, go back and forth... Instead, in the private facilities, it’s easier, you can go there as soon as you are ready and do the screening. It’s just this. Then, when it comes to everything else, everything is well organised”*

(woman, 51 years old, employee, colorectal screening).

*“The population could be helped by offering not only mammograms but also ultrasound as a screening exam, which is not foreseen beyond age 46. I asked to have it because the doctor said it is an age in which people are more prone to problems”*

(woman, 49 years old, teacher, breast screening).

Furthermore, the experience respondents had before, during, and after the screening was influenced by whether—and in which ways—the health providers accepted or rejected their concerns:

*“She was fantastic, I mean, welcoming, detailed, very precise, which is pretty crucial. The communication and the relationship between the practitioner and patient are essential… at that moment, I was her patient”*

(woman, 51 years old, nurse, colorectal screening).

*“The doctor who did my mammogram was very nice, kind, and did not make me feel uncomfortable at all. On the contrary, some doctors make you feel uncomfortable. The doctor here was exceptional”*

(woman, 62 years old, housewife, breast screening).

#### 3.2.4. The Affective Determinants of Cancer Screening Participation

This macro-category derives from the emerging sense of belonging and collective identity of southern Italians, which was relevant among our participants. The affective connotation of the choices regarding cancer screenings in Campania highlights the identity and belonging factor that binds people to certain places and certain healthcare workers.

This macro-category comprises two categories: The role of the facility and The role of health providers.

Based on our interviews, the adhesion to the screening campaigns proposed by the public Campania healthcare seems largely emotionally connoted. From the analysis of the texts, among those invited to the screening, the ones who decided to adhere did so because they were affectively attached to the facility or the health providers working there. Such affective attachment is meant as a bond, a previous history, or an experience that inspired trust and a sense of security. Many women who have undergone screening reported they gave birth in that same facility or have known its practitioners for years because they lived nearby and visited that place many times. Others, on the other hand, stated they went there because of the good reputation of a particular doctor who they respected and trusted, or even because a colleague or friend worked at the facility and got them involved. This suggests the existence of a close link between being familiar with the facility and the doctors, a sense of trust and security, and actual adherence to the cancer screening programs:

*“The Annunziata is the facility par excellence, I always say that it is the place where lives are born. I always said that.. because lives were born there, I used to bring babies here. For me, this place represents trust”*

(woman, 55 years old, school collaborator, breast screening) (the Annunziata hospital was founded in the 14th century as an institution for the care of abandoned children, and was an important point of reference for Neapolitans for the care of children and their mothers).

*“Apart from the fact that I have a sister who is a doctor, my husband is a scientific informant, and I always knew, I can’t tell you how, but I always knew that there were these check-ups which needed to be done. Furthermore, between friends, it is said: ‘Why don’t you go to the ASL* (an *Azienda Sanitaria Locale* (ASL) is a public authority within the Italian public administration, responsible for providing public health services in a specific territory, usually provincial)*? You can join the women’s prevention programs; you don’t need to pay anything because you are over a certain age…’*

(woman, 62 years old, housewife, breast screening).

*“Thanks to my brother-in-law, I did the ultrasound here”*

(woman, 58 years old, employed, breast screening).

#### 3.2.5. Partners and Children

This macro-category is deeply linked to the emerged core category Family and Familiarity. In fact, having children or having a partner is indeed a central element in the choice of whether or not to have checks and prevention. The value attributed to life and being “healthy” finds meaning in the presence of someone to take care of. Prevention is thus not mainly self-care, but it reflects caring for others, and through the care of others participants are able to actually take care of themselves. According to our participants, to “be healthy” is a key element because it enables them to take care of others, mainly their partners or children.

This macro-category includes two categories: Prevention and children and Prevention and couples.

Prevention is perceived not only as an act of care towards oneself, but primarily as taking care of others. Women, in particular, associate the need to perform preventive actions with the desire to exercise their parental authority to guarantee their children a healthy mother who can take care of them:

*“I have a 10-year-old girl, I had her when I was 40. I want to see her grown up, so I am afraid because of the familiarity with diseases”*

(woman, 50 years old, employed, breast screening).

*“I have a child, and I want to see him grow up because I grew up without a mother, so I know precisely what it’s like. I want to be next to my child as long as possible, so I must be very careful. I always have to be vigilant, avoiding bad things, because prevention is better than cure!”*

(woman, 27 years old, cervical screening).

Many of those who decided to adhere to the screenings and to pursue a healthy lifestyle do so not only out of a sense of responsibility towards their children, but also towards their partner and, more in general, their family:

*“Because if I neglected myself, I would abandon my two children, my husband, my parents who would suffer a lot… Well, also for myself, because I would prefer to stay alive a little longer”*

(woman, 51 years old, housewife, breast screening).

*“What would I do with my children? How could I not think about them?”*

(woman, 49 years old, housewife, breast screening).

#### 3.2.6. Physical Sensations and Emotions in the Course of Action

When facing the screening exam, women describe their emotions by differentiating the sensations they experience before, during, and after (while waiting for the results) the screening. The modesty of exposing the body and the anxiety of uncertainty are some of the aspects collected in the coding.

This macro-category includes four categories: Emotions before the screening, Emotions during the screening, Emotions after the screening, and Physical sensations.

While thinking about the moment of the screening, the interviewees described experiencing various emotions, such as anxiety about possible discomfort or pain. In the case of mammography and cervical screening, the prevailing physical sensation is pain. After the examination, the fear of discovering a disease predominates. Attending the screening at a public facility implies a long wait before getting the exam results. Such a wait can last up to thirty days, during which anxiety and fear are intense. For this reason, many people state they prefer to attend these exams among private healthcare, but not everyone can afford this choice:

*“I always feel a little anxious until the results arrive. I am always thinking about it. There is always the fear of bad news. But then I wait anyway...”*

(woman, 54 years old, housewife, cervical screening).

*“Well, those of waiting will be difficult days, full of anxiety. There’s also the hope that no phone call will arrive…”*

(woman, 49 years old, housewife, cervical screening).

*“I was afraid to do it, I was quite worried about the idea of feeling the pain, but once I was there, I faced it. The moment I was facing it, I said to myself: ‘Ok, I’ve done. Now I just have to wait for the results’”*

(woman, 54 years old, lawyer, breast screening).

*“Pain, when he squeezed my breast in that thing, yes. This guy was good compared to the one I found last time I came here, who just threw it there… Instead, he assisted me really well, and, in fact, I told him he was very good”*

(woman, 56 years old, office worker, breast screening).

#### 3.2.7. Protective Actions

Our interviewees describe the strategies implemented to counteract the negative emotions and sensations they felt before, during, and after screening. Starting from the codes that emerged, we created the macro-category: protective actions.

This macro-category encompasses three categories: Healthy behaviours, Spirituality, and Preventive actions.

While facing the fear of illness and death, as well as family stories of suffering, our interviewees stated they activate some protective actions through which they feel they are protecting themselves from possible risks. As for our interviewees, engaging in prevention does not mean avoiding the chances of contracting the disease, but helps gain precious time to treat it. In contrast, in our participants’ opinion, the actions that can be responsible for reducing the risks of illness are those that can stimulate self-love: good nutrition, physical activity, quitting smoking, implementing beauty routines, and, in general, caring for personal mental health—which was particularly affected by the COVID-19 pandemic. Notably, the codes good nutrition and physical activity have the highest frequencies. This attests that our interviewees strongly share the idea that carrying out these actions has a protective function in counteracting the onset of future diseases:

*“It’s hard to say, but I’m 55 years old. I try to have a correct diet, everyone makes sacrifices, but I also try to do some physical activity. I stopped smoking a while ago, so I think having a more or less correct diet and doing physical activity allows me to live my life optimistically”*

(man, 55 years old, office worker, colorectal screening).

*“I follow a diet. First of all, I do physical activity. I’m a smoker. I’m trying to quit, to smoke less and just get rid of this habit because it’s so unhealthy. In the meantime, I also get informed about the check-ups suggested for my age. To feel safe and physically healthy, I do everything possible”*

(woman, 27 years old, unemployed, cervical screening).

*“I take care of my external health because that is also important. If I care, I like to do my scrubs, nice hot baths, masks…”*

(woman, 56 years old, housewife, breast screening).

Another interesting element emerging from the analyses is religious faith, intended as a protection capable of saving from illness and giving peace to the soul when worries, conflicts, anxiety, or stress appear:

*“I’m always afraid of receiving bad news, so sometimes I don’t sleep so well at night. I have bad dreams, I dream that it’s not going well, but then I think that Jesus will help me, and I hope it will go well. And thank God everything has gone well for me in the last two years”*

(woman, 51 years old, housewife, breast screening).

*“Well… It occurs to me that we are in the hands of the Lord. What God wants will happen”*

(woman, 64 years old, secretary, breast screening).

#### 3.2.8. Promotion and Dissemination

How the information about screening procedures is provided was an important theme in the narratives of our interviewees, who reiterated that they had sometimes made explicit the difficulties encountered. Our interviewees particularly emphasised how a specific action and/or adequate information can be decisive in carrying out or foregoing screening. According to them, information would be welcomed positively if it was shared among groups of peers or doctors who have a connection with the individual or their family.

This macro-category encompasses two categories: Information and Actions for the community.

The research participants mainly attribute low cancer screening attendance rates to a lack of information among citizens. Indeed, most of them learned about the free screening programs from doctors they privately knew or friends who had already joined. Thus, word of mouth seems to be the main source of dissemination of such information.

According to our respondents, an effective way to better spread the information about these programs would be to convey campaigns via social media, to be addressed to people of all ages so that younger people can raise awareness among their families:

*“Let’s say that the healthcare system should accommodate prevention and inform people more, get them more involved, because maybe because of a lack of information or for the high cost of so many examinations, many people do not succeed, don’t do it, in short. But, in my opinion, there should be a strong campaign precisely targeting prevention because, in any case, the diffusion of cancer is increasing and the diseases are many, so the national healthcare system is responsible for all of them”*

(man, 55 years old, employee, colorectal screening).

In addition, our interviewees also suggested other concrete actions on a community level, proposing themselves as testimonials for the service. Moreover, they advocate the importance of reducing bureaucracy and making the fruition of the service more direct, as is the case with travelling trucks:

*“I would tell my friends that undergoing the screening is like nothing. It’s not painful at all or, at least, if it is painful, it is just a matter of a few minutes. People just have to do it”*

(woman, 42, architect, breast screening).

*“I had the opportunity to know about the screenings thanks to the travelling truck that the region organised. They came to the town where I work”*

(woman, 53 years old, teacher, breast screening).

*“I do prevention within the private healthcare system, first of all because it consents more immediate access. Here [at the public facilities], I have to accept the specialist on duty, whom, for goodness’ sake, I greatly trust, but do not know personally”*

(woman, 61, employed, cervical screening).

### 3.3. The Core Category. Family and Familiarity: Prevention as an Act of Care for Others

Cancer, disease, and risk appear to be associated with two words: family history. The fears, representations, anxieties, and behaviours enacted are linked to participants’ own accounts of events, losses, and illnesses in the family. These experiences have the power to activate a subjective and individual ways of experiencing prevention in general, and cancer screenings in particular.

Treatment, prevention, and adherence to the screenings are acts that generate anxiety and fear, which, however, turns into a sense of security and trust if, on the other side of the desk, people find a person who is familiar to them, or a person able to appear welcoming and make them feel somewhat at home.

Most often, those who come to undergo the screening do so because they have been accompanied by a family member or a loved one, because they have been urged by a doctor they know, or because they are familiar with the facility, which they trust and have been attending for years.

Familiarity has a dual meaning. On the one hand, it can be intended as both a genetic or psychological familiarity with cancer and other diseases, meaning that people can feel more or less at risk of developing the disease according to whether their direct familiars had contracted it, but also that just knowing someone who faced such illness can make it closer in their perception. On the other hand, it can also refer to the intimacy and friendliness that users can find in the health providers, which alleviates fears related to the actual moment of the screening and the possible outcomes.

In light of these findings, the development of intervention programs or general guidelines to increase the adherence to cancer screening programs in Campania should capitalise on these observations by enhancing or creating spaces of safety and familiarity that activate the sense of trust in people who do not habitually refer to a public facility, and for those who live in fear due to genetic heredity or just a previous vicarious experience with cancer.

Moreover, the other element that emerged strongly from the interviews concerns the well-diffuse representation of taking care of oneself in order to take care of others. The desire to be healthy mainly stems from the need to care for one’s own children, partner, and family. Subsequently, interventions aimed at increasing cancer screening attendance should build on this feeling of collective responsibility, with campaigns involving the whole family and not just the individual for itself.

In this way, socially connotating the concrete actions to be implemented on the regional administrative level would make it possible to shape and define new ways of acting and thinking about cancer prevention and treatment at both the institutional and individual levels. This would result in a sharper link between the institutional and public health objectives, and the key issue of a general lack of motivation on the individual and social level, for addressing which the main tools should, therefore, be health education and citizen participation.

## 4. Discussion

The present study aimed to qualitatively investigate the experiences and meanings that users of cancer screening services freely offered within the public healthcare system attributed to prevention and participation in the screening program itself.

The material collected through interviews confirmed that just thinking about undergoing the screening exam can arouse strong emotions, often close to fear and anxiety. In particular, feelings such as fear of discovering a disease, concern about procedures, the doctors, or even just entering a hospital or clinic, emerged. As counterintuitive as it might appear, our findings suggest that for a healthy woman or man, the stress of medical check-ups is significant enough to determine the avoidance of the appointment [19]. Alongside fear, shame is another affective determinant of avoidant behaviour [20].

By definition, screening programs target people showing no sign of the disease. Still, gathering information is crucial for making choices dictated by awareness instead of anxiety and fear. The literature studying the link between fear of cancer and engagement in treatment and prevention pathways offers many insights in this regard [21,22]. On the one hand, the fear of developing the disease can be the driving force behind the actual enacting of requesting appointments with doctors and having regular check-ups. On the other hand, all of the more or less active avoidance behaviours can also be attributed to fear. This phenomenon has been studied in the peculiar context of cancer screenings [9]. For example, in the case of cervical screening, one of the most-referred reasons for women adhering to screening programs is precisely the fear of the disease and the perception of the screening as a routine examination [23]. At the same time, the women who refuse to be tested fear the medical exams and the health providers most, or who perceive themselves as healthy, do not feel the need to be screened. This is partly consistent with what has been studied concerning colorectal cancer screening, for which fear, embarrassment, and disgust about the screening procedure are the main emotional barriers to participation [24].

Furthermore, the literature on cancer prevention highlights the role of information as a possible antidote to anxiety [25,26], and as something that can increase adherence rates [27]. In the case of cancer screening, the first step is deeply understanding the importance of participation by considering the purpose of the exam and the procedures through which tests are performed. For instance, many citizens do not know that these medical exams are generally slightly or not invasive at all, and can definitely be considered life-saving when performed regularly. In addition, the findings from the current study also suggest that the most determining factor is not the information itself, but rather the *recognition* given to the person providing the information.

The participants claimed to have received correct information several times, but this was not enough to prompt them to adhere to the screenings. Rather, those who finally decided to act were mainly driven by the emotional closeness and authority attributed to the person who suggested they participate in the screenings.

According to the interviews, a further meaningful area concerns waiting for results. After undergoing the cancer screening exam, the procedure imposes waiting to receive the results, sometimes for weeks. This time can become too long in participants’ perception, during which anxiety, fear, worry, and sometimes even distress and sadness tend to be high. Respondents claimed to know that there is no *rational* motivation for the many negative thoughts. Still, it is precisely the fear of the unknown that triggers thoughts about the worst possible scenario. Hence, participants affirmed the need either to reduce the waiting time or to implement strategies that help people spend that time suspending their judgment and avoiding feeding those fears.

Finally, a significant and innovative result reached by this study concerns the importance of the other in individual prevention practices. Participants, in fact, reported they could only cope with the distress related to undergoing the screenings thanks to the presence of their significant others. As a matter of fact, many of them ended up doing it because they were accompanying a friend or supporting a family member. In light of these findings, we can hypothesise that cancer screening programs might focus primarily on the idea of caring for those you love rather than caring for yourself. By adopting this perspective, there is a shift from focusing on an individual dimension of care to a community approach, according to which resistances are overcome through the presence of the other, who acts as a mirror and makes it possible to approach personal health effectively.

This concept of caring for loved people led to the definition of the core category, namely Family and Familiarity. Prevention can, therefore, be read as an act of caring for others, and, in these terms, fear and embarrassment are mitigated by the presence of another person, as an act of reciprocal care, in which the individual cares for him or herself through the care of another whom he or she feels close, and who makes them feel welcomed in the practice of prevention by buffering the most negative thoughts and emotions. In this reading, the other functions as a protective element that allows individuals to deal with an issue that would otherwise be perceived as impossible to handle. In addition, becoming familiar with the facility and the health providers stimulates feelings of trust and safety that prompt people to overcome avoidant conduct and actively participate in cancer screening programs.

## 5. Limitations

The current study employs a qualitative methodology and, consequently, does not have the generalisability of the results as its ultimate aim. In addition, as the survey is part of a wider project focused on promoting adhesion to cancer screening in the Italian Campania region (MIRIADE), the study goals explicitly target the population living in this territory. Therefore, we cannot exclude that the core category that emerged, Family and Familiarity, indicates the specific way of approaching health that characterises such sociocultural context. Therefore, future investigations should address this topic from a cross-cultural perspective, also in a gender approach, and employ mixed methods to detail which findings are shared and which differentiate the Campania population from others.

Moreover, according to our objectives, we chose to include in the study only people who were already using cancer screening services offered within the public healthcare system to be able to understand their motivations for participating, as well as which resistance they had in the past or have to deal with every time they undergo a screening exam. However, the results of this study could be further enriched by collecting the experiences and testimonies of people who do not visit public facilities (e.g., those who prefer to undergo prevention exams at facilities that operate within the private healthcare system) and people who do not participate at all in screening programs despite knowing about it.

Notwithstanding these limitations and the future perspectives opened up by the study, our results offer interesting insights that could orienteer future campaigns promoting cancer screening programs, particularly within public healthcare. According to our analyses, campaigns promoting these services should focus primarily on the feeling of collective responsibility, with programs involving the entire household and not only the individual.

## 6. Conclusions

The current study contributes to the psychosocial literature on cancer screening participation, offering valuable insights into how individuals perceive their participation in these medical exams, with some practical implications for enhancing cancer screening initiatives. Our primary finding is well expressed by the emerged core category, defined as Family and Familiarity. This concept essentially deals with two considerations. First, individuals’ ways of experiencing health prevention in general, and cancer screenings in particular, are strictly linked with their personal and familiar history with both the illness and the practical and emotional assistance offered by the healthcare professionals. This emphasizes the importance for healthcare providers to prioritize the human relationship with the users of the screening services, with particular attention to their individual characteristics and specific needs. Medicine of the future has to be centred on gathering individual experiences. It should not theorize through generalizations, but be ready to embrace the singularities present in each user. Fear, anxiety, shame and uncertainty are not static theorizing, but have infinite meanings for each individual, as emerged from our research. Fear is not only an emotion, but it gathers the memory of past experiences, the bodily memory of the first time it was experienced, with all the related physical and mental sensations. On this consideration relies our idea, according to which future guidelines on cancer screenings in Campania should take into consideration the individual and cultural representations that the screenings themselves evoke.

Coherently, the second meaning conveyed by the emerged core category refers to the specific ways in which Campania citizens view health. Precisely, our data suggest that it is considered a precious personal asset, but also that it is often pursued to protect loved ones from the potential suffering and practical difficulties associated with one’s illness rather than as a tool for achieving personal well-being. These insights shed light on the complex interplay between personal health motivations and considerations for the well-being of one’s close relationships, enriching our understanding of the broader landscape of the determinants of health behaviours. Future interventions aimed at increasing cancer screening attendance in this population should thus take into account the role of the familiars and the social network in general as potential effective drivers to individual participation in screening initiatives. Furthermore, future research should deepen our knowledge about this altruistic component of the motivation to attend to personal health by employing both qualitative and quantitative methodology and by recruiting individuals who participate, as well as those who do not participate, in cancer screening programs.

## Figures and Tables

**Table 1:** Participants’ characteristics.

Gender	N	%
Male	7	6.8
Female	96	93.2
**Offspring**		
Yes	88	85.4
No	15	14.6
**Work Status**		
Housewife	25	24.5
Teacher	12	11.8
Lawyer	3	2.9
Health professional	7	6.9
Manager	2	2.0
Architect	1	1.0
Librarian	1	1.0
Office worker	25	24.5
Secretary	4	3.9
Social manager	1	1.0
Hairdresser	1	1.0
Retired	6	5.9
Unemployed	15	13.7
**Family History of Cancer**		
Yes	55	53.4
No	48	46.6
**Preferred Healthcare Services**		
Only public healthcare facilities	57	55.3
Mainly public healthcare facilities (with some sporadic appointments in private services)	46	44.7

**Table 2:** Categories and macro-categories.

Category	Macro-Category
1. Representation of health	Health and Body
2. Body representation	
3. Family stories	Relationship with Cancer and Other Diseases
4. Individual stories	
5. Stories of loved ones	
6. Positive aspects	Health Facilities and Health Providers
7. Negative aspects	
8. Public healthcare vs. private healthcare	
9. The role of the facility	The Affective Determinants of Cancer Screening Participation
10. The role of health providers	
11. Prevention and children	Partners and Children
12. Prevention and couples	
13. Emotions before the screening	Physical Sensations and Emotions in the Course of Action
14. Emotions during the screening	
15. Emotions after the screening	
16. Physical sensations	
17. Healthy behaviours	Protective Actions
18. Spirituality	
19. Preventive actions	
20. Information	Promotion and Dissemination
21. Actions for the community	

## Data Availability

The data that support the findings of this study are available from the corresponding author upon reasonable request.
